# Effect of Endocrine Disruptor Pesticides: A Review

**DOI:** 10.3390/ijerph8062265

**Published:** 2011-06-17

**Authors:** Wissem Mnif, Aziza Ibn Hadj Hassine, Aicha Bouaziz, Aghleb Bartegi, Olivier Thomas, Benoit Roig

**Affiliations:** 1 Laboratoire de Biochimie, Unité de Recherche 02/UR/09-01, Institut Supérieur de Biotechnologie, de Monastir, BP 74, 5019 Monastir, Tunisia; E-Mails: w_mnif@yahoo.fr (W.M.); aziza.hadjhassine@gmail.com (A.I.H.H); w_bouaziz.aicha@yahoo.fr (A.B.);; 2 Institut Supérieur de Biotechnologie de Sidi Thabet, Pole Technologie Sidi Thabet, 2020 Ariana, Tunisia; 3 Department of Biology, Faculty of Sciences, King Faisal University, P.O. Box 1759, 31982, Al Hassa, Saudi Arabia; E-Mail: bartagi_fsm@yahoo.com; 4 Environment and Health Research laboratory (LERES), Advanced School of Public Health (EHESP), Avenue du Professeur Léon Bernard - CS 74312, 35043 Rennes Cedex, France; E-Mail: olivier.thomas@ehesp.fr (O.T.)

**Keywords:** endocrine disruptors, pesticides, biomonitoring, human effect

## Abstract

Endocrine disrupting chemicals (EDC) are compounds that alter the normal functioning of the endocrine system of both wildlife and humans. A huge number of chemicals have been identified as endocrine disruptors, among them several pesticides. Pesticides are used to kill unwanted organisms in crops, public areas, homes and gardens, and parasites in medicine. Human are exposed to pesticides due to their occupations or through dietary and environmental exposure (water, soil, air). For several years, there have been enquiries about the impact of environmental factors on the occurrence of human pathologies. This paper reviews the current knowledge of the potential impacts of endocrine disruptor pesticides on human health.

## 1. Introduction

Since the discovery of DDT in 1939 [1], numerous pesticides (organochlorides, organophosphates, carbamates) have been developed and used extensively worldwide with few guidelines or restrictions. In industrialized countries, the Green Revolution of the 1960s significantly increased agricultural productivity by increasing the cultivated surfaces, mechanization, planting of hybrid crops with higher yields, and pest control [2]. This fight requires the massive use of pesticides, which are hazardous chemicals designed to repel or kill rodents, fungi, insects, and “weeds” that undermine intensive farming. The main effects of pesticides represent a great benefit for human health. Indeed, they help control agricultural pests (including diseases and weeds) and plant disease vectors, human and livestock disease vectors and nuisance organisms, and organisms that harm other human activities and structures (gardens, recreational areas, *etc*.). Moreover, they insure increased food production, a safe and secure food supply, and other secondary benefits [3]. However, many first generation pesticides have been found to be harmful to the environment. Some of them can persist in soils and aquatic sediments, bioconcentrate in the tissues of invertebrates and vertebrates, move up trophic chains, and affect top predators.

Rachel Carson’s book “*Silent Spring*”, published in 1962 [4], first drew attention to the hazard of the widespread extensive use of pesticides for the environment (namely birds) and also for human health. The book resulted in big modifications to the US national policy on pesticides, leading to a national ban on DDT and certain other pesticides.

Worldwide consumption of pesticides for agricultural use is constantly increasing, rising from 0.49 kg/ha in 1961 to 2 kg/ha in 2004 (see various web sources, such as for example http://ec.europa.eu/agriculture/envir/report/fr/pest_fr/report.htm#fig6; http://faostat.fao.org/site/424/default.aspx#ancor; http://www.goodplanet.info/eng/Food-Agriculture/Pesticides/Pesticides/(theme/266)), and humans and wildlife are today continuously exposed to a number of pesticides via the environment (surface water, ground water, soil), food and drinking water [5].

The World Health Organization (WHO) has reported that roughly three million pesticide poisonings occur annually, resulting in 220,000 deaths worldwide [6]. In some cases, it has been suggested that diseases such as cancer, allergies, neurological disorders and reproductive disorders may be connected to pesticide exposure.

This article focuses on pesticides that act as endocrine disruptors, and reviews the available information about the exposure and effects of such pesticides, as well as human biomonitoring for human health risk assessment.

## 2. Effects of Endocrine Disruptor Pesticides

Many chemicals that have been identified as endocrine disruptors are pesticides [7–11]. About 105 substances can be listed, and most of them are shown in Table 1. Of these, 46% are insecticides, 21% herbicides and 31% fungicides; some of them were withdrawn from general use many years ago but are still found in the environment (ex. DDT and atrazine in several countries).

EDCs act mainly by interfering with natural hormones because of their strong potential to bind to estrogen or androgen receptors [12] as shown in Table 1. In particular, EDCs can bind to and activate various hormone receptors (AR, ER, AhR, PXR, CAR, ERR) and then mimic the natural hormone’s action (agonist action). EDCs may also bind to these receptors without activating them. This antagonist action blocks the receptors and inhibits their action. Finally, EDCs may also interfere with the synthesis, transport, metabolism and elimination of hormones, thereby decreasing the concentration of natural hormones. For example, thyroid hormone production can be inhibited by some ten endocrine disruptor pesticides (amitrole, cyhalothrin, fipronil, ioxynil, maneb, mancozeb, pentachloronitro-benzene, prodiamine, pyrimethanil, thiazopyr, ziram, zineb, not shown in Table 1) [13–16].

At the environmental level, wildlife is particularly vulnerable to the endocrine disrupting effects of pesticides. Effects linked to endocrine disruption have been largely noted in invertebrates [17–21], reptiles [22–27], fish [28,29], birds [30–34] and mammals [35–38] as reviewed by Mnif *et al*. [39]. Most of them are linked to exposure to organochlorine pesticides (OC) and affect the reproductive function. For example, a study on *Daphnia magna* has shown that endosulfan sulphate disrupts the ecdysteroidal system (regulating processes such as molting and embryonic development) and juvenile hormone activity (regulating the sex ratio) of crustaceans [40,41]. Another example is the influence of linuron on reproductive hormone production [42], testosterone production in rats being significantly reduced after in utero exposure to linuron, whereas progesterone production was not affected [42].

At the human level, endocrine disruptor pesticides have also been shown to disrupt reproductive and sexual development, and these effects seem to depend on several factors, including gender, age, diet, and occupation.

Age is a particularly sensitive factor. Human fetuses, infants and children show greater susceptibility than adults [43–45]. Much of the damage caused by EDC occurs during gametogenesis and the early development of the fetus [45–48]. However, the effects may not become apparent until adulthood. Moreover, fetuses and infants receive greater doses due to the mobilization of maternal fat reserves during pregnancy [47–50] and breastfeeding [49,51]. Infants are extremely vulnerable to pre and postnatal exposure to endocrine disruptor pesticides, resulting in a wide range of adverse health effects including possible long-term impacts on intellectual function [52,53] and delayed effects on the central nervous system functioning [54,55].

Likewise, residential proximity to agricultural activity is a factor often described to explain developmental abnormalities in epidemiological studies of low birth weight [56], fetal death [57], and childhood cancers [58]. Additionally, a higher prevalence of cryptorchidism and hypospadias [59,60] was found in areas with extensive farming and pesticide use and in sons of women working as gardeners [61]. Recently, a relation has been reported between cryptorchidism and persistent pesticide concentration in maternal breast milk [47,62,63]. The impact of endocrine disruptor pesticides on fertility has also been discussed [64].

Based on the epidemiological studies since 2000, the study concluded that pesticide exposure may affect spermatogenesis leading to poor semen quality and reduced male fertility. Furthermore, an increasing number of epidemiological studies tend to link environmental exposure to pesticides and hormone-dependent cancer risks. High levels of PCBs, DDE, and DDT have been found in fat samples from women with breast cancer [141]. The risk of breast cancer is said to be four times greater in women with increased blood levels of DDE 142]. One of the latest epidemiological studies performed in Spain between 1999 and 2009 shows that among a total of 2,661 cases of breast cancer reported in the female population, 2,173 (81%) were observed in areas of high pesticide contamination [143]. Moreover, it was also suggested that women with hormone responsive breast cancer have a higher DDE body burden than women with benign breast disease [144]. Similar studies have revealed correlations between damage to the immune system and increased amounts of organochlorine residues in certain cancerous tissues [145]. Numerous other studies support the hypothesis that pesticide exposure influences the risk of breast cancer [146], but few of them are really conclusive due to some inconsistent data across the study. Further research is required to explore long-term follow-up beginning in early life, with opportunities for exposure measurement at critical periods of vulnerability. Moreover, improvements are needed in the cohort sample size and standardization of exposure assessments methods. Finally, researchers also need to consider simultaneous co-exposures to these substances and other chemicals and whether they may act in an additive, synergistic, or antagonistic manner [147].

There may also be a connection between pesticide exposure and prostate cancer. Various studies have consistently demonstrated a higher risk in agricultural populations than in the general population [148–150]. For example, pesticides (in particular DDT) were associated with a statistically significant higher rate of prostate cancer among farmers (exposed to organochloride pesticides) in a multi-site case-control study carried out in five rural areas between 1990–92 in Italy [151]. Several studies in the USA and Sweden showed that farmers and commercial pesticide applicators have a slightly and/or significantly higher rate of prostate cancer than the general population [148,152,153].

Several meta-analyses, cohort studies and case-control studies on the risk of prostate cancer in populations exposed occupationally or professionally to pesticides have been conducted in recent years [154] (and reference therein). They all showed a significantly higher risk of prostate cancer estimated at between 10 and 40%, the higher values being for professional exposure. Quite recently, a study analyzed the relationship between exposure to chlordecone (organochloride pesticide extensively used for more than 30 years in the French West Indies to control the banana root borer) and the risk of prostate cancer [155]. It showed a significant increase in the risk of prostate cancer with increasing plasma chlordecone concentrations and supported the hypothesis that exposure to environmental estrogens may increase the risk of prostate cancer.

However, in spite of these outcomes, the hypothesis that such excess risk is related to the use of pesticides has not yet been formally demonstrated. Various other factors have been suggested to explain the increase in prostate cancer in agricultural or rural populations, such as dietary issues, contact with infectious agents via livestock, dust, tobacco and chemical products [154]. Rigorous studies with larger cases that accurately and objectively estimate pesticide exposures and consider gene-environment interactions are needed to determine a potential relationship between pesticides and prostate cancer.

## 3. Biomonitoring for Human Exposure Assessment

Exposure to pesticides can occur via numerous pathways, including household use of pesticide products, dietary exposure to pesticide residues, and exposure to agricultural drift. Biological monitoring studies indicate that pesticide exposures are widespread in the human population. Dietary exposure comes from residues in fruits, vegetables, and from contaminated meat, fish, rice and dairy products. The European Commission [156] estimated that, in 2005, consumer intake was always below the acceptable daily intakes (ADI) for long-term exposures. Several recent studies also show the difference between EDI and ADI [157–159]. However, according to the European report, the acute reference dose (a parameter for high short-term intakes, usually in one day or one meal) was exceeded for some pesticides in different vegetables and fruits and 26.7% of samples show residues of more than one pesticide, with a significant upward trend as compared to previous years. Food intake is not the only exposure pathway for the general population. Living near sites where pesticides are used, manufactured or disposed of may significantly increase environmental exposure through inhalation and contact with air, water and soil [160–163].

Human exposure to pesticides is assessed by measuring the levels of pesticides in human samples such as breast milk, maternal blood and serum, urine and sometime umbilical cord blood. Improvements in analytical techniques have made it possible to detect pesticides and their metabolites at trace levels (from milligrams per kilogram to femtograms per kilogram in some laboratories) in almost all human samples. Table 1 reports the detection of pesticides in human samples.

Most of these studies show evidence of higher levels of pesticides in the exposed population (for example due to their occupation or geographical location) than in non-exposed control people. For example, a relation was established between employment in agriculture of Spanish women during pregnancy and serum levels of organochlorine endocrine disruptor pesticides, including DDT and isomers (despite their being banned in Spain since 1977) [164]. Also, higher levels of DDTs and HCHs were found in maternal milk and blood samples in Chinese provinces than in developed or industrialized countries [110]. Finally, higher concentrations of several pesticides were also found in urine and plasma of pregnant Israeli women compared to other populations of pregnant women in the United States and the Netherlands.

Pesticide metabolites are also monitored in human samples because they can be representative of a global contamination. This is particularly true for organophosphorus compounds. Alkyl phosphates have been reported in human samples (urine, hair) as representative of exposure to organophosphate pesticides [165–168], and some authors have observed a significant difference in the levels of total dialkyl phosphates among exposed and no exposed groups.

## 4. Discussion and Perspectives

Endocrine disruptor pesticides are widely used for agricultural, municipal, home and medical purposes worldwide. Humans are exposed to these compounds, and due to their toxic properties, the consequences of this exposure on human hormoel-dependent pathologies are being established. Most risk assessment studies and some epidemiologic studies have looked at the exposure and toxicology of a single compound. However, two other considerations must be included: the presence of pesticide by-products and the cumulative exposure to pesticides multiresidue.

It is undisputed that in some cases, pesticide by-products can exhibit greater harmful effects than their parent compounds. For example, one study showed that, at the organism level, the only sublethal effect seen was an increase in heart rate at low concentration and a decrease at higher concentration with the use of aldicarb-sulfoxide but not with aldicarb [169]. Another study reported that the oxons of methyl-parathion, chlorpyrifos and diazinon were 15 to 10 times more toxic (to sperm DNA) than their corresponding parent compounds [170]. As another example, *in vitro* studies confirmed that 2,4-dichlorophenoxyacetic acid (2,4-D), a commonly used organophosphate herbicide promoting the proliferation of androgen-sensitive cells [171], is a known estrogen receptor ligand [172]. Vinclozolin degrades to several metabolites in the soil, in the plants and in animal organisms [173]. Two hydrolytic degradation products, 2-[[(3,5- dichlorophenyl)-carbamoyl]oxy]-2-methyl-3-butenoic acid and 3′,5″-dichloro-2-hydroxy-methylbut-3-enalide, have been identified as anti-androgenic compounds that mediate the adverse effects of vinclozolin [173].

Furthermore, the combined actions of pesticides also need also to be addressed in the risk assessment process because mixtures of these substances may cause higher toxic effects than those expected from the single compounds [174]. For example an equimolar mixture of three pesticides (deltamethrin, methiocarb, and prochloraz) suppressed androgen receptor (AR) activation *in vitro* [175]. Also, under the additional presence of simazin and tribenuronmethyl, weight changes of the adrenal gland and alterations in gene expression of AR-associated genes were observed *in vivo* in castrated testosterone-treated rats. The question of the combined effect of mixtures of contaminants has attracted the attention of the scientific community. Predictive approaches are generally based on the mathematical concepts of Concentration Addition (CA) and Independent Action (IA), both predicting the toxicity of a mixture based on the individual toxicities of the mixture components (e.g., [176] and references therein). Recently, a review showed that some other models could be useful as tools to assess combined tissue doses and to help predict potential interactions including thresholds for such effects [177].

Finally, the impact of synthetic pesticides, due in particular to an excessive use (including environmental pollution and implications to human health) have led to modifications in agricultural practices and various national and international regulations limiting their use. Further limitations and/or bans should be sought, along with alternative solutions that are safer and non-toxic to the environment and humans. One such alternative is so called “natural pesticides” that are not synthetically produced, but are derived from nature such as botanicals pesticides (pyrethrum, limonene, and many others), microbial/biological agents (microbes, parasites) and inorganic minerals (boric acid, limestone, diatomaceous earth). These solutions are generally assumed to be less toxic for human health than synthetic pesticides and could represent an interesting alternative. But their usefulness is actually questionable because some such pesticides are not potent enough to control pests but at the same time do exhibit adverse effects for human health (i.e. “natural pyrethroids”). Further studies are needed on the occurrence, fate and impact of such pesticides on the ecosystem and public health.

## Figures and Tables

**Table 1.** Effects of different groups of endocrine disruptorpesticides and their chemical structures (adapted from [65]).

**Pesticides** [66]	**Chemical structure**	Endocrine Disruptor **Effects**	**Biomonitoring in human samples**
**2,4-D (H)** M(g/Mol) = 221 pKa = 2.73 logP: 2.81	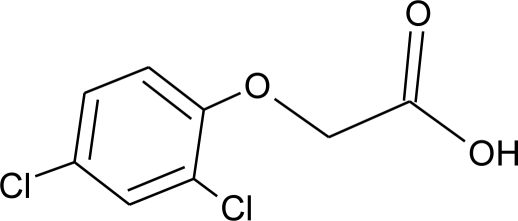	Synergistic androgenic effects when combined with testosterone [67]	U: <LOD–598 ng/mL [68] S : 0.07–0.56 μg/g creatinine [69]
**Acephate (I)** M(g/Mol) = 183.2 pKa = 8.35 logP: −0.85	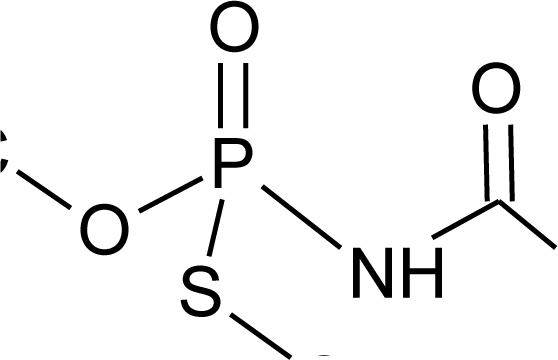	Disruption of hormone expression in the hypothalamus [70]	U: <LOD–0.26 ng/mL [68] H.S: 7.2 μg/mL [71] **
**Acetochlor (H)** M(g/Mol) = 269.8 pKa = n.a logP: 4.14	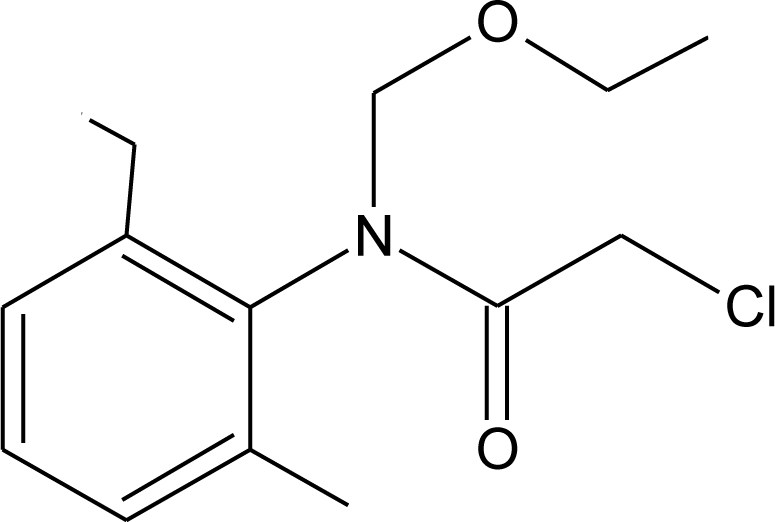	Interaction with uterine estrogen receptors, alteration of thyroid hormone dependant gene expression [72,73]	U: <LOD–10.9 ng/mL [68] S: 0.08–0.10 μg/g creatinine [69]
**Alachlor (H)** M(g/Mol) = 269.8 pKa = 0.62 logP: 3.09	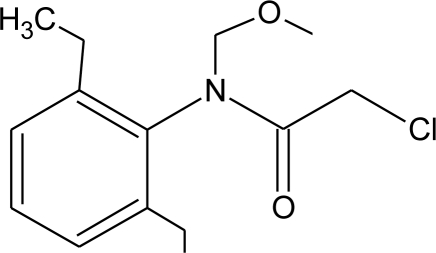	Competitive binding to estrogen and progesterone receptors. Interaction with the pregnane X cellular receptor, interfering with the production of enzymes responsible for steroid hormone metabolism [13,74]	U: <LOD–305 ng/mL [68] S: 0.31–0.72 μg/g creatinine [69]
**Aldicarb (I)** M(g/Mol) = 190.3 pKa = n.a logP: 1.15	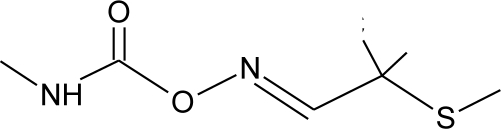	Inhibition of 17 beta-estradiol and progesterone activity [13,75]	
**Aldrin (I)** M(g/Mol) = 364.9 pKa = n.a logP: 6.5	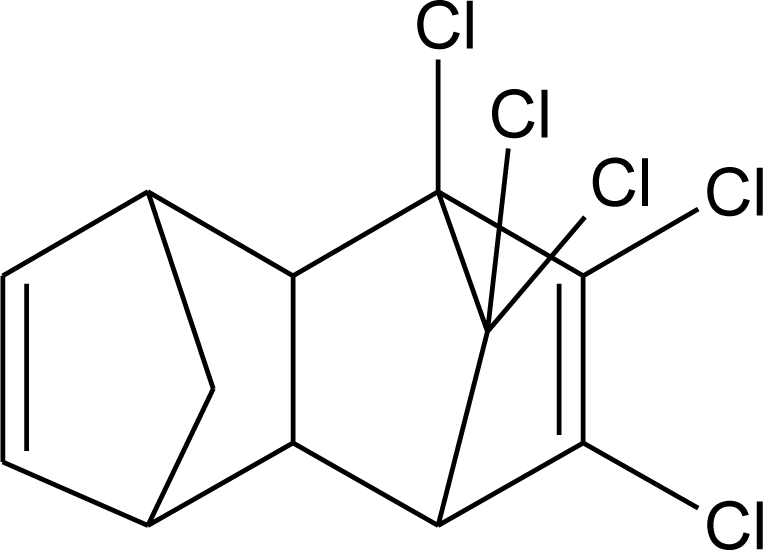	Competitive binding to androgen receptors [76]	H.S: 2.17–372 μg/L [77,78] H.M: mean 0.03 mg/L ± 0.03 [79] A.T: 25.6–137.2 ng/g lipid [78]
**Atrazine (H)** M(g/Mol) = 215.7 PKa = 4.14, 10.7 logP: −0.97	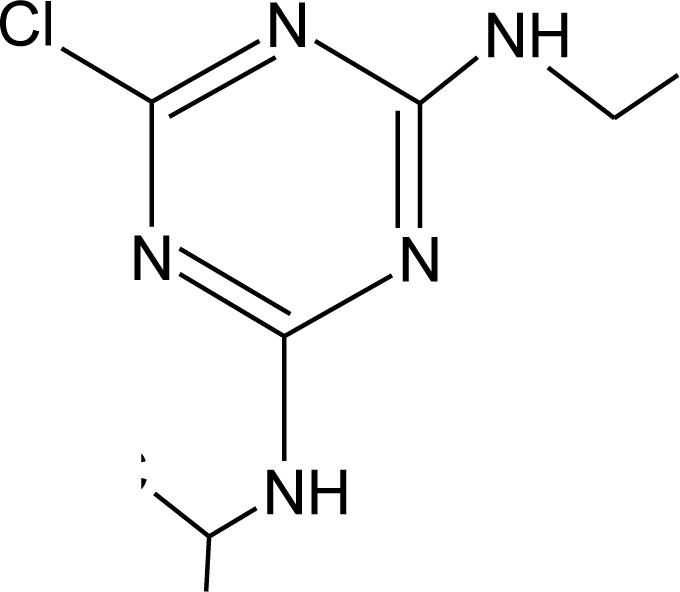	Androgen inhibition, weak estrogenic effect. Disruption of the hypothalamic control of lutenising hormone and prolactin levels. Induction of aromatase activity, increase estrogen production. Adrenal glands damages and reduction of steroid hormone metabolism [13,80–83]	U: <LOD–9.2 ng/mL [68,84] H.S: mean 2 pg/g [76,85] S: 0.07–0.17 μg/g creatinine [69]
**Bendiocarb (I)** M(g/Mol) = 223.2 pKa = 8.8 logP: 1.7	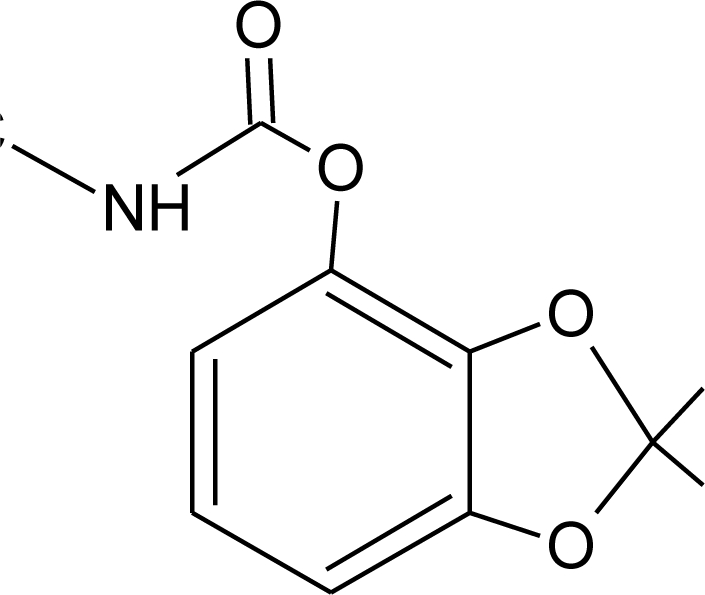	Weak estrogen effect [13]	
**Benomyl (F)** M(g/Mol) = 290.3 pKa = 4.48 logP: 1.4	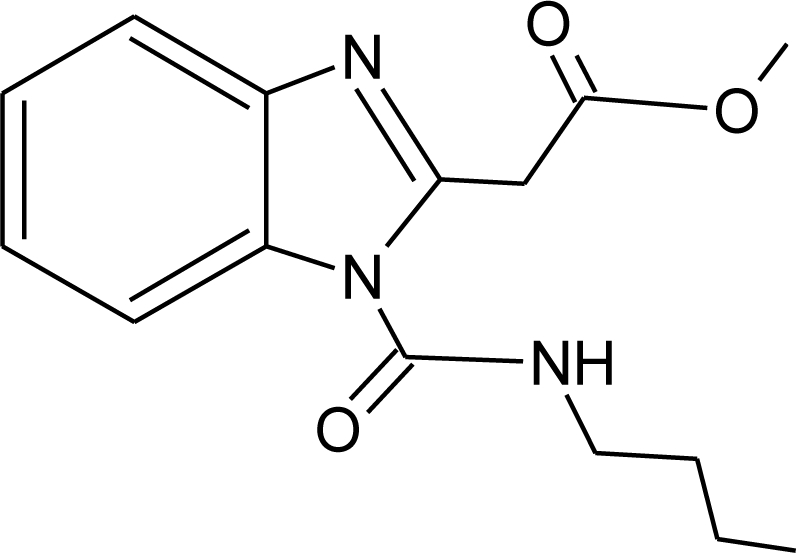	Increase of estrogen production and aromatase activity [86]	
**Bioallethrin (I)** M(g/Mol) = 302.4 pKa = n.a logP: 4.68	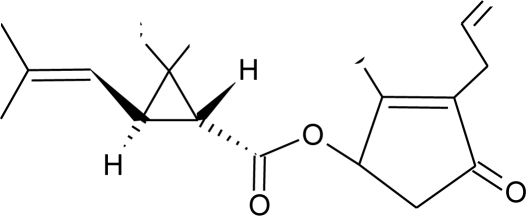	Inhibition of estrogen-sensitive cells proliferation [87]	M: 0.61–1.79 μg/mL [88] H: 1.08–2.74 μg/mL [88]
**Bitertanol (F)** M(g/Mol) = 337.4 pKa = n.a logP: 4.1	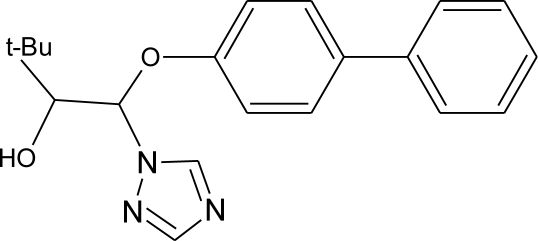	Inhibition of aromatase activity, decrease of estrogens production and increase of androgens availability [89]	
**Bupirimate (F)** M(g/Mol) = 316.4 pKa = 4.4 logP: 3.68	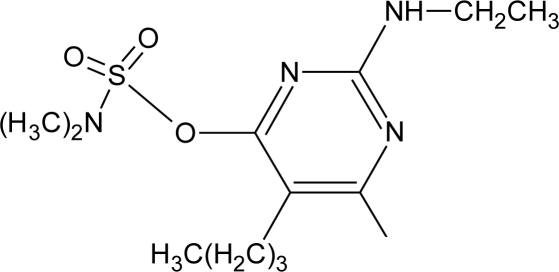	Activation of Pregnane X cellular receptor [11]	
**Captan (F)** M(g/Mol) = 300.6 pKa = n.a logP: 2.5	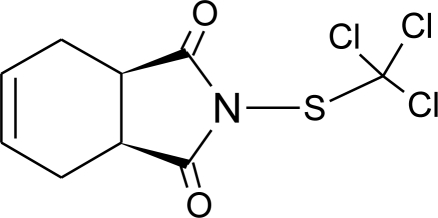	Inhibition of estrogen action [90]	
**Carbaryl (I)** M(g/Mol) = 201.2 pKa = 10.4 logP: 2.36	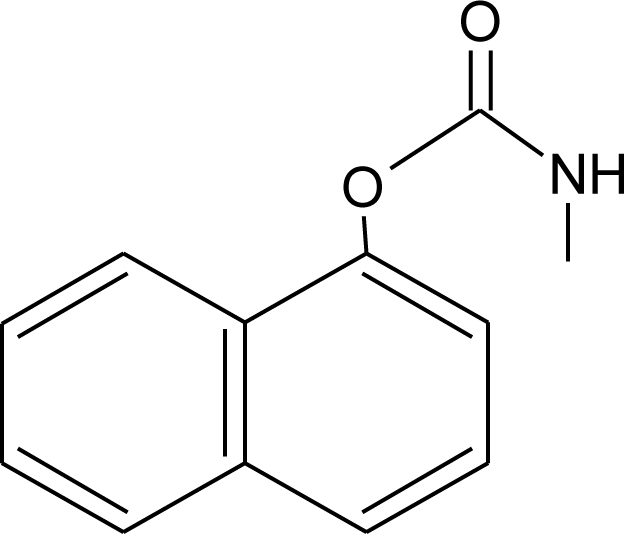	Weak estrogen effect [13]	
**Carbendazim (F)** M(g/Mol) = 191.2 pKa = 4.2 logP: 1.48	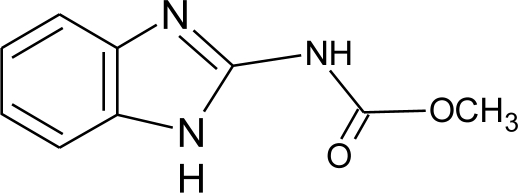	Increase of estrogen production and aromatase activity [86]	
**Carbofuran (I)** M(g/Mol) = 221.2 pKa = n.a logP: 1.8	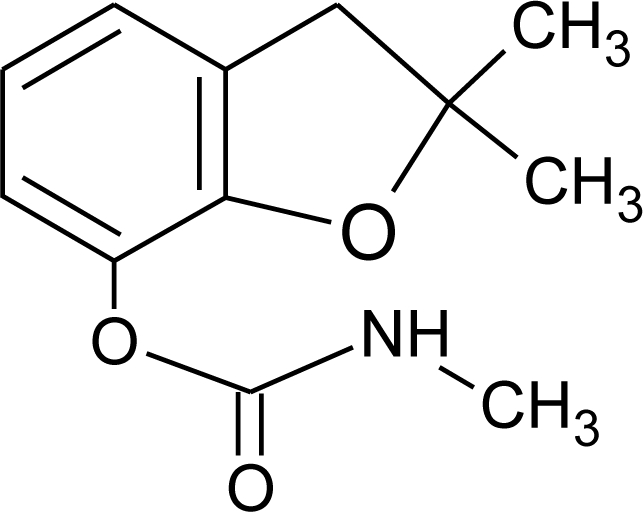	Increase of progesterone, cortisol and estradiol level and decrease of testosterone one [91]	M.S: 0.007–17.63 ng/g [92] U.C: 0.007–13.97 ng/g [92]
**Chlorothalonil (F)** M(g/Mol) = 265.9 pKa = n.a logP: 2.94	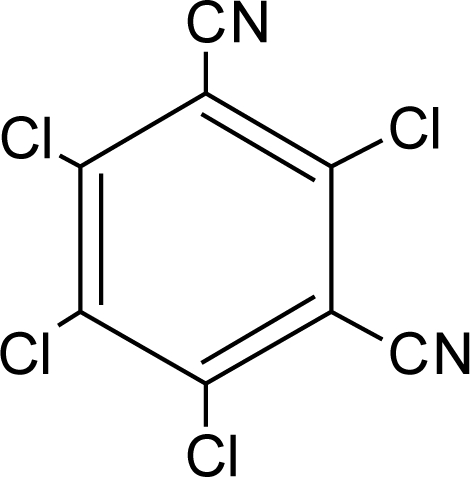	Activation of androgen-sensitive cells proliferation [93]	M.S: 0.007–25.31 ng/g [92] U.C: 0.007–25.12 ng/g [92] H.S: mean 6 pg/g [85]
**Chlordane (I)** M(g/Mol) = 409.8 pKa = n.a logP: 2.78	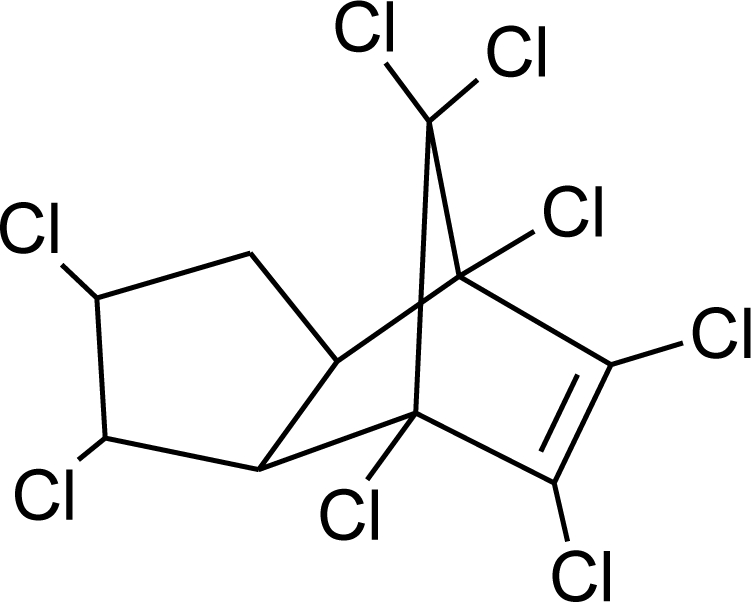	Competitive binding to androgen receptors [76] Anti-estrogenic effect, inhibition of estradiol binding [13]	M.P: 0–2.7 ng/g lipid [94] B.S: <LOD–0.9 ng/g lipid [95] H.M: 0.02–437 ng/g lipid [79,96–99] FF: 0.1–0.3 ng/L [100]
**Chlordecone (I)** M(g/Mol) = 490.6 pKa = n.a logP: 4.5	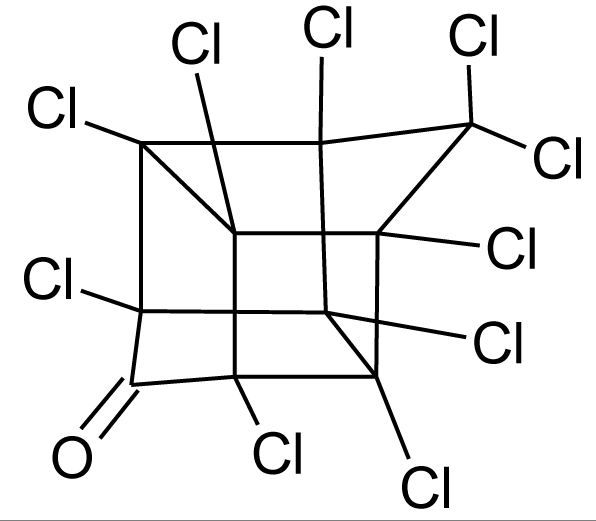	Binding to estrogen and androgen receptors [90,101,102]	
**Chlorfenviphos** (I) M(g/Mol) = 359.6 pKa = n.a logP: 1.36	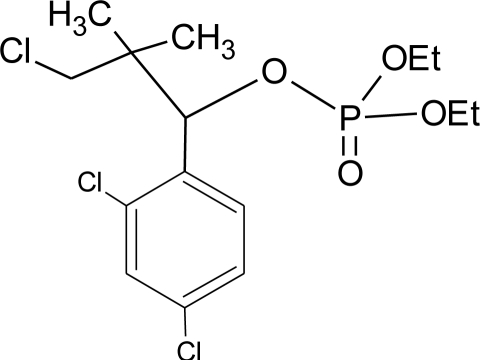	Weak estrogen effect [103]	
**Chlorpyrifos methyl (I)** M(g/Mol) = 322.5 pKa = n.a logP: 4	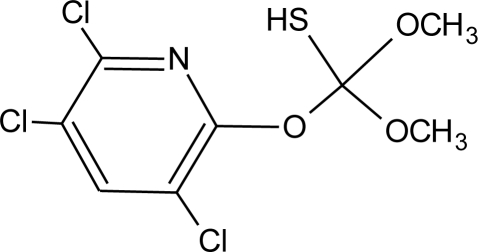	Antagonist to androgen activity [104]	U : <LOD–57.7 ng/mL* [68,84] M.S: 0.0007–10.1 ng/g [92] U.C: 0.0007–1.84 ng/g [92] H.S: mean 9 pg/g [85] H: 1.77–2.16 1.83 μg/mL [88]
**Cypermethrin (I)** M(g/Mol) = 416.3 pKa = n.a logP: 5.3	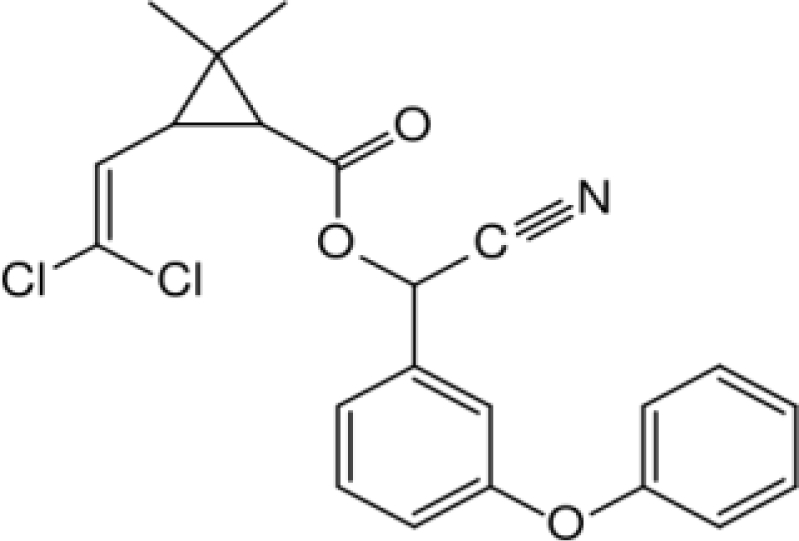	Estrogenic effect [105,106]	U: 0.5–100.4 μg/g * [107] M: 1.85–2.43 μg/mL [88]
**Cyproconazole (F)** M(g/Mol) = 291.8 pKa = n.a logP:3.09	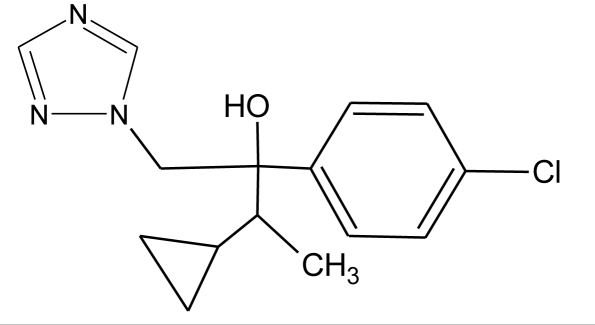	Inhibition of aromatase activity, decrease of estrogens production and increase of androgens availability [89]	
**DDT and metabolites (I)** M(g/Mol) = 354.5 pKa = n.a logP:6.91	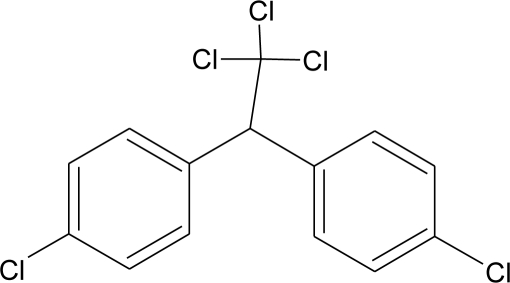	Competitive binding to androgen receptors, activation of androgen-sensitive cells proliferation. Stimulation of estrogen receptor production, estrogen receptor agonist and PR antagonist [76,93,108,109]	M.P: 0.2–3588 ng/g lipid [94] B.S: <LOD–40.9 ng/g lipid [95] HM: 3.9–4700 ng/g lipid [110,96–99,111] A.F: 0.1–0.63 mg/L [112] M: 1.1–2.8 μg/mL [88] H: 0.17–0.65 μg/mL [88,113] M.B: 0–6168 ng/g lipid [110,114] H.S: 12.5–814.9 ng/mL [77] U.C: 189–3296 ng/g lipid [110]
**Deltamethrin (I)** M(g/Mol) = 505.2 pKa = n.a logP: 4.6	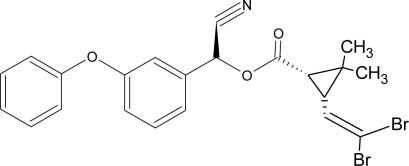	Weak estrogenic activity [8]	U: 0.5–57.7 μg/g * [107]
**Diazinon (I)** M(g/Mol) = 304.4 pKa = 2.6 logP: 3.69	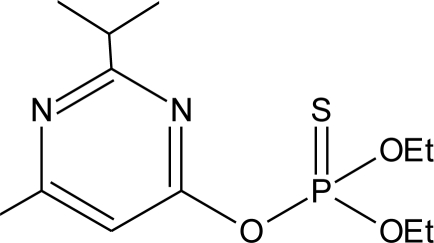	Estrogenic effect [115]	S: 1.84–4.96 μg/g creatinine * [69] H.S: mean 2 pg/g [85]
**Dichlorvos (I)** M(g/Mol) = 221 pKa = n.a logP: 1.9	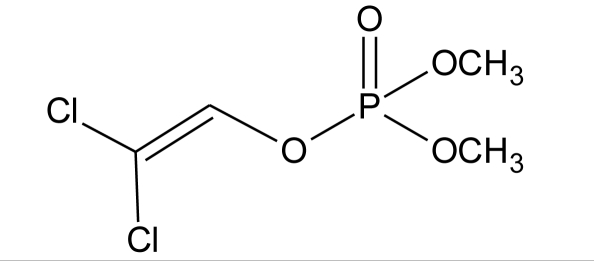	Weak androgen-receptor antagonist [8]	
**Dicofol (I)** M(g/Mol) = 370.5 pKa = n.a logP: 4.3	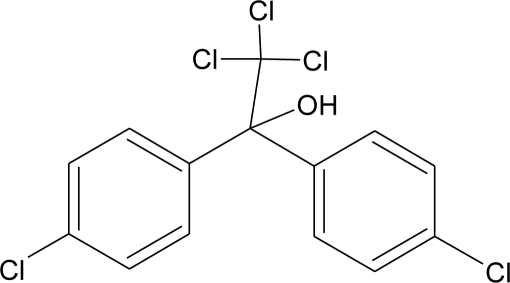	Inhibition of androgen synthesis, increase of estrogens synthesis, binding to estrogen receptor [90,83]	
**Dieldrin (I)** M(g/Mol) = 380.9 pKa = n.a logP: 3.7	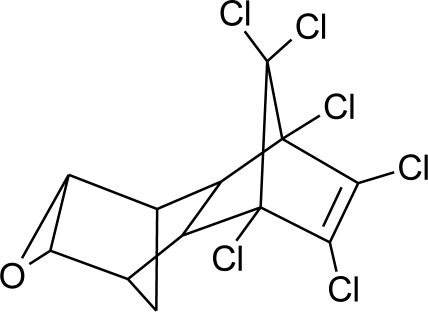	Competitive binding to androgen receptors, estrogenic effect, stimulation of estrogen receptor production [8,76,108,116]	H.M: <0.1–64 ng/g lipid [111]
**Diflubenzuron (I)** M(g/Mol) = 310.7 pKa = n.a logP: 3.89	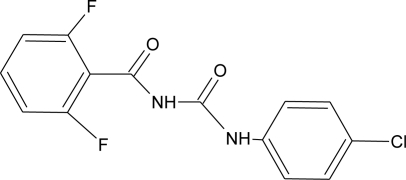	Pregnane X cellular receptor activation [11]	H.S: 1.21–356.4 μg/L [77,78] H.M: mean 0.66 mg/L ± 1.75 [79] A.T: 17.01–84.05 [78]
**Dimethoate (I)** M(g/Mol) = 229.3 pKa = n.a logP: 0.704	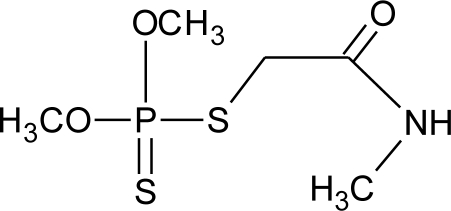	Disruption of thyroid hormones action. Increase of insulin blood concentration, decrease of luteinizing hormone blood concentration [117,118]	
**Diuron (H)** M(g/Mol) = 233.1 pKa = n.a logP: 2.87	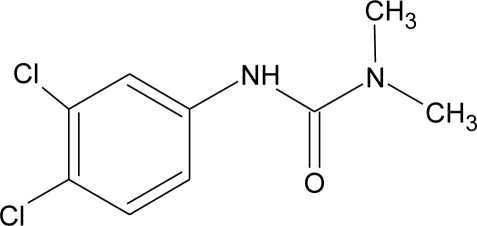	Inhibition of androgens action [83]	
**Endosulfan (I)** M(g/Mol) = 406.9 pKa = n.a logP: 4.75	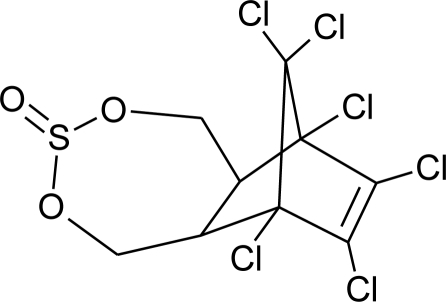	Competitive binding to androgen receptors, estrogenic effect, stimulation of estrogen receptor production, inhibition of aromatase activity [8,76,109,116]	H.S: 8.85 547.6 μg/L [77,78] A.T: 21.4 417.6 ng/g lipid [78]
**Endrin (I)** M(g/Mol) = 380.9 pKa = n.a logP: 3.2	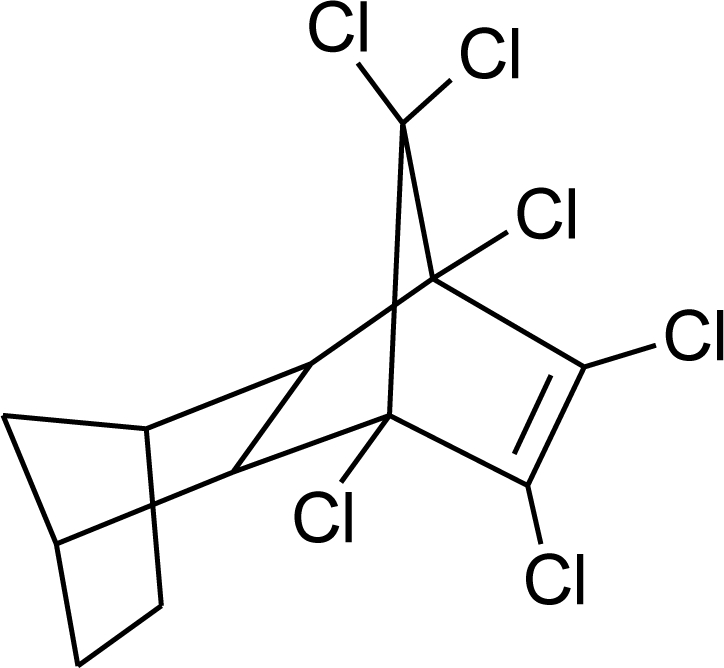	Competitive binding to androgen receptors [76]	H.M: mean 0.65 mg/L ± 1.63 [79] H.S: 1.21 6.35 μg/L [78] A.T: 47.43 148.13 ng/g lipid [78]
**Epoxyconazole (F)** M(g/Mol) = 329.8 pKa = n.a logP: 3.3	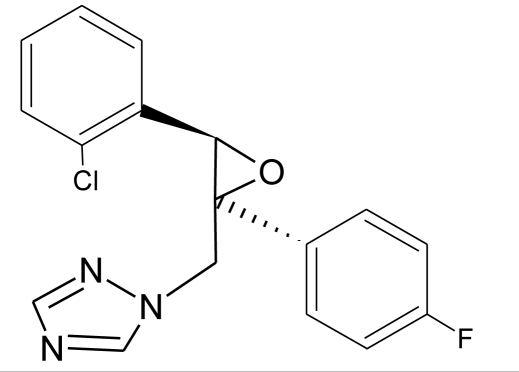	Inhibition of aromatase activity, decrease of estrogen production and increase available androgens [89,119]	
**Fenarimol (F)** M(g/Mol) = 331.2 pKa = n.a logP: 3.69	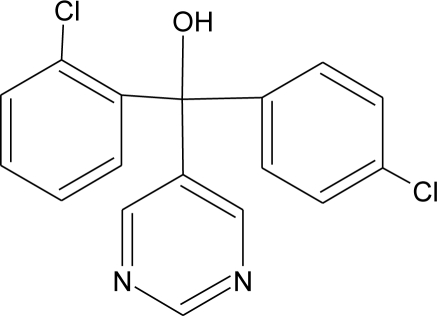	Antagonist of androgenic action. Potential aromatase inhibition. Pregnane X cellular receptor activation [8,11,120]	
**Fenbuconazole (F)** M(g/Mol) = 336.8 pKa = n.a logP: 3.79	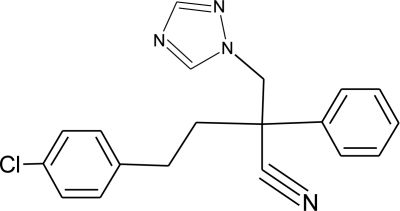	Inhibition of thyroid hormones production, Pregnane X cellular receptor activation [11,13]	
**Fenitrothion (I)** M(g/Mol) = 277.2 pKa = n.a logP: 3.32	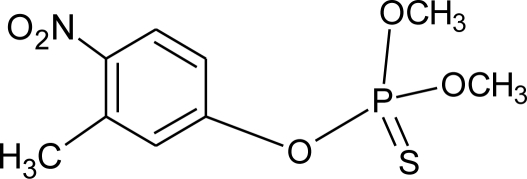	Competitive binding to androgen receptor, inhibition of estrogens action [90,121]	H.S: 4.5 μg/mL [71] **
**Fenoxycarb (I)** M(g/Mol) = 301.3 pKa = n.a logP: 4.07	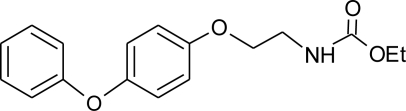	Interference with testosterone metabolism [122]	
**Fenvalerate (I)** M(g/Mol) = 419.9 pKa = n.a logP: 5.01	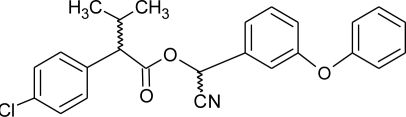	Inhibition of estrogen-sensitive cells proliferation, antagonist of the progesterone action [86,123]	
**Fluvalinate (I)** M(g/Mol) = 502.9 pKa = n.a logP: 3.85	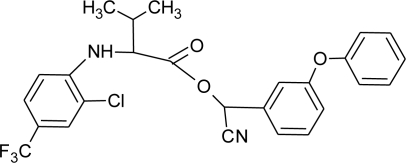	Binding to human sex hormone, Inhibition of progesterone production [124,125]	
**Flusilazole (F)** M(g/Mol) = 315.4 pKa = 2.5 logP: 3.87	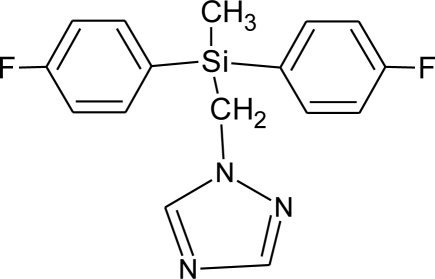	Inhibition of aromatase activity, decrease of estrogens production, increase of available androgens [89]	
**Flutriafol (F)** M(g/Mol) = 301.3 pKa = 2.3 logP: 2.3	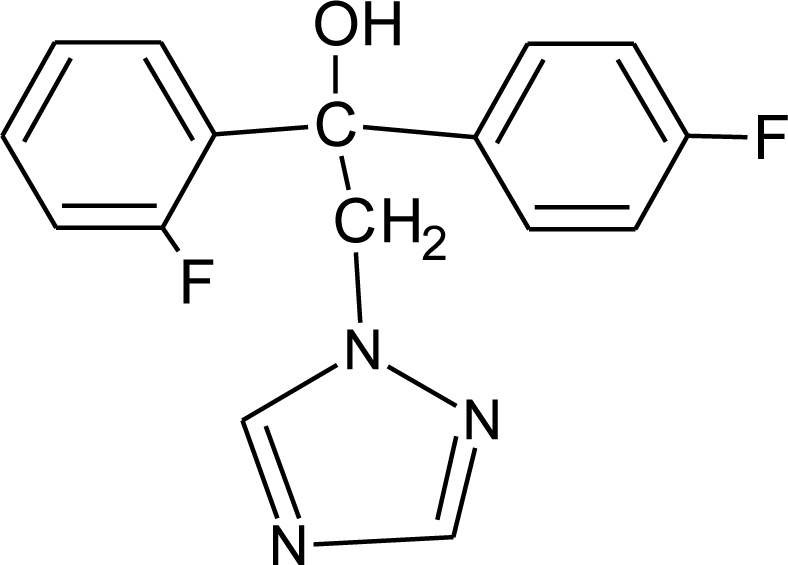	Weak estrogen inhibition [119]	
**Glyphosphate (H)** M(g/Mol) = 168.1 pKa = 0.78; 2.34; 5.96; 10.98 logP: −3.2	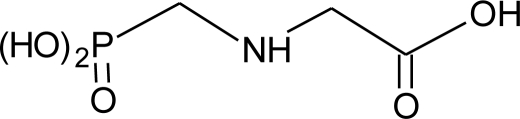	Disruption of aromatase activity, preventing the production of estrogens [126]	U: 1.1–2.1 ng/mL [84]
**HCB (F)** M(g/Mol) = 284.8 pKa = n.a logP: 3.93	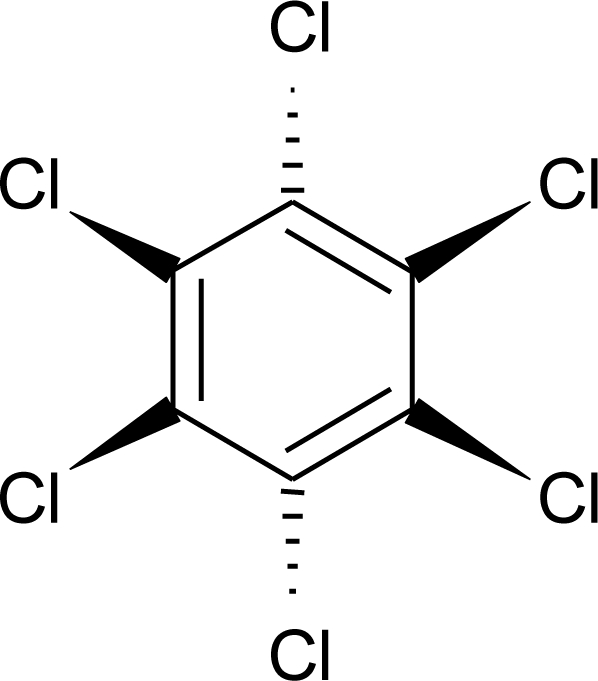	Severely disruption of thyroid hormone production. Enhancement of androgen action at low doses, but inhibition at high levels [127,128]	M.P: 1.6–44.3 ng/g lipid [94] B.S: 7.4–37.2 ng/g lipid [95] H.M: 0.4–472 ng/g lipid [79,96–99] H.S: 12.5–393.3 μg/L [77] H 1.2–15.9 pg/mg [113] F.F: 0.11–0.2 ng/L [100] A.F: [112]
**HCH (lindane) (I)** M(g/Mol) = 290.8 pKa = n.a logP: 3.61	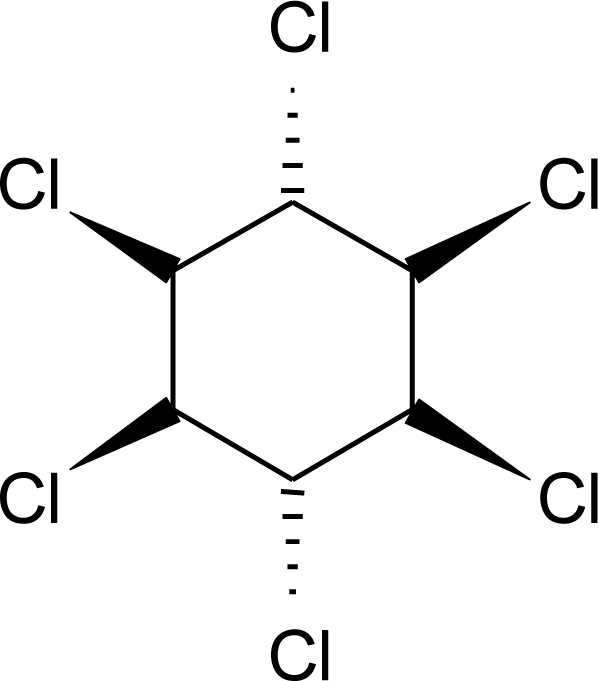	Reduction of oestrous cycles and luteal progesterone concentrations. Increase of insulin and estradiol blood serum concentrations, decrease thyroxine concentrations. Competitive binding to AR, ER and PR [117,129]	M.P: 0.4–2839 ng/g lipid [94] B.S: <LOD–134 ng/g lipid [95] H.M: 4.7–8700 ng/g lipid [80,110,96–99] H.S: 1.08–265.8 μg/L [77,78] H: 50.7–235 pg/mg [113] A.T: 17.44–113.31 ng/g lipid [78] M.B: 1.9–386.6 ng/g lipid [110, 114] A.F: 0.1–0.26 ng/mL [112] U.C: 4–130 ng/g lipid [110]
**Heptachlor (I)** M(g/Mol) = 373.3 pKa = n.a logP: 5.44	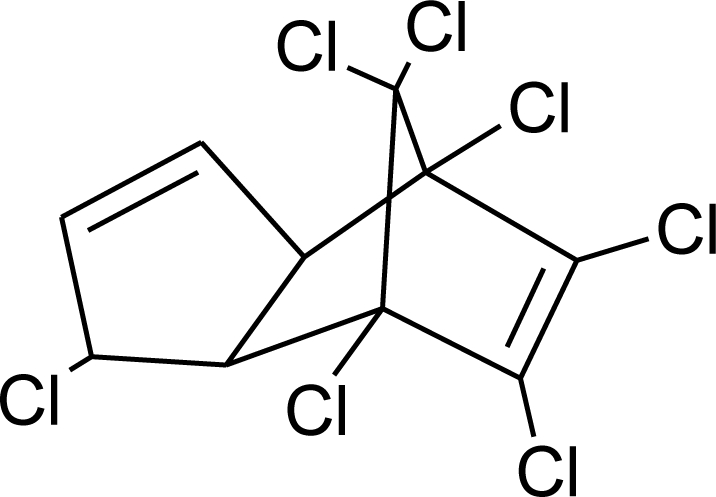	Binding to cellular estrogen and androgen receptors [130,131]	M.P: 0.2–5.2 ng/g lipid [94] B.S: <LOD–0.9 ng/g lipid [95] Human serum: 12.5–139.1 μg/L [77] H.M: mean 0.07 mg/L ± 0.34 [79]
**Hexaconazole (F)** M(g/Mol) = 314.2 pKa = 2.3 logP: 3.9	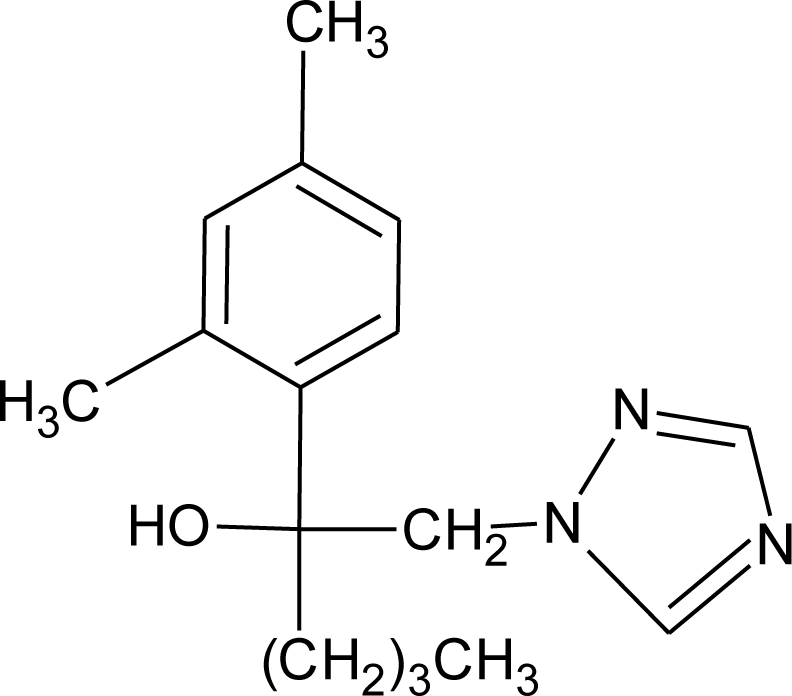	Inhibition of aromatase activity, decrease of the estrogens production and increase of available androgens [89]	
**Isoproturon (H)** M(g/Mol) = 206.3 pKa = n.a logP: 2.5	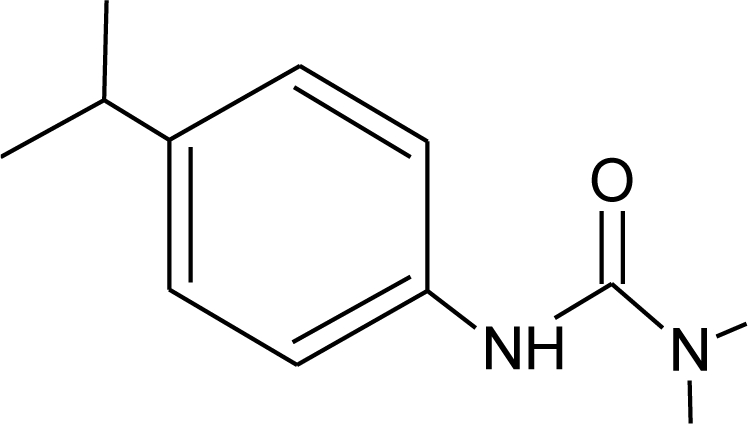	Pregnane X cellular receptor activation [11]	
**Iprodione (F)** M(g/Mol) = 330.2 pKa = n.a logP: 3.1	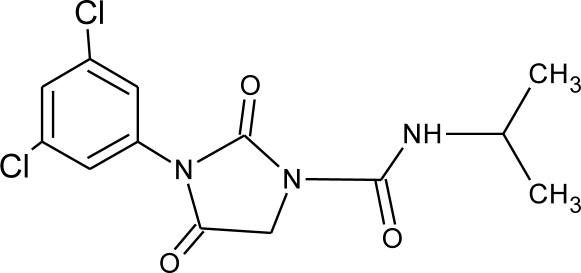	Increase weakly aromatase activity, and estrogen production [8]	
**Linuron (H)** M(g/Mol) = 249.1 pKa = n.a logP: 3	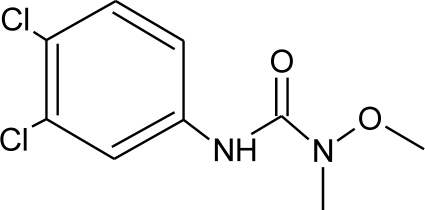	Competitive binding to androgen receptor, thyroid receptor agonist [131,132]	
**Malathion (I)** M(g/Mol) = 330.4 pKa = n.a logP: 2.75	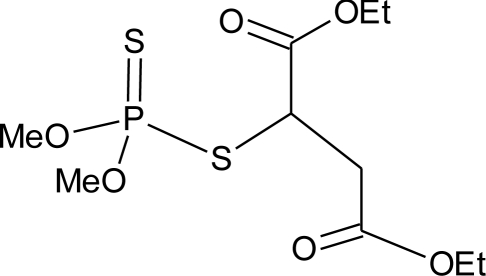	Inhibition of catecholamine secretion, binding to thyroid hormone receptors [13,133]	U: <LOD–3195 ng/mL [68] * M: 2.92–5.38 μg/mL [88] H: 1.62–2.12 μg/mL [88] S: 0.37–0.92 μg/g creatinine [69]
**Methiocarb (H)** M(g/Mol) = 225.3 pKa = n.a logP: 3.18	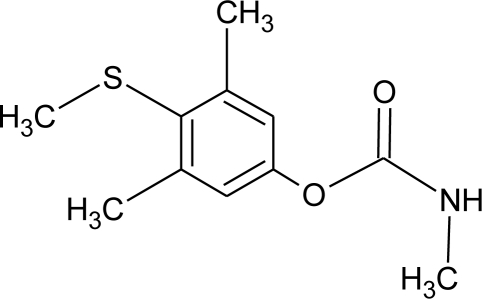	Inhibition of androgen activity and increase of estrogen one [8]	
**Methomyl (I)** M(g/Mol) = 162.2 pKa = n.a logP: 1.24	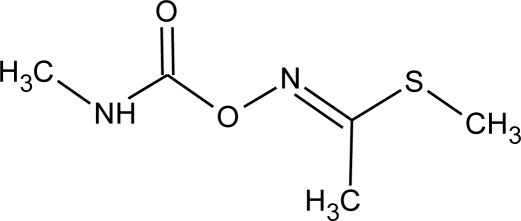	Weak increase of aromatase activity and estrogen production [8,13]	
**Methoxychlor (I)** M(g/Mol) = 345.7 pKa = n.a logP: 5.83	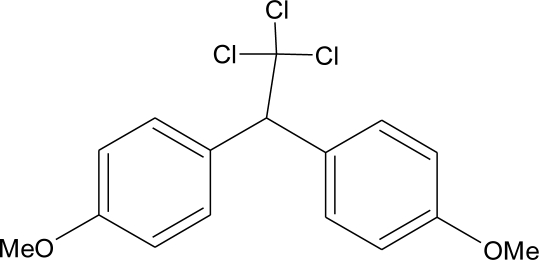	Strong estrogenic effect. Competitive binding to androgen receptor, interaction with the pregnane X cellular receptor [13,74,76]	H.S: 0.38–0.39 μg/L [78] A.T: 29.86–155.58 ng/g lipid [78]
**Metolachlor** M(g/Mol) = 283.8 pKa = n.a logP: 3.4	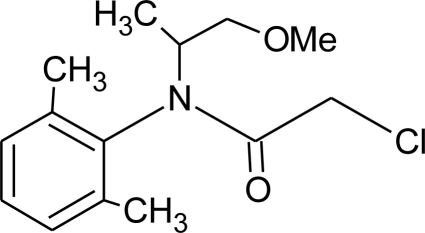	Pregnane X cellular receptor activation [11]	U: <LOD–4.5 ng/mL [68,84] H.S: mean 2 pg/g [85] M.S: 0.007–1.96 ng/g [92] U.C: 0.007–2.37 ng/g [92] S: 0.20–0.48 μg/g creatinine [69]
**Metribuzin (H)** M(g/Mol) = 214.3 pKa = 0.99 logP: 1.65	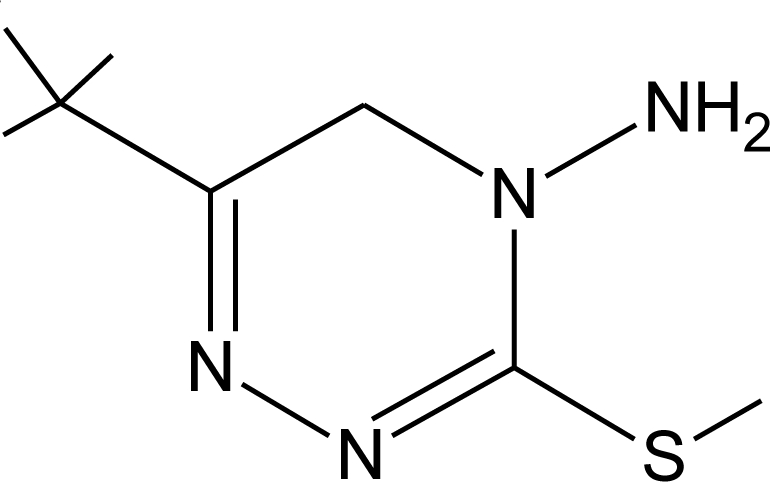	Hyperthyroidism, alteration of somatotropin levels [134]	
**Mirex (I)** M(g/Mol) = 545.5 pKa = n.a logP: 5.28	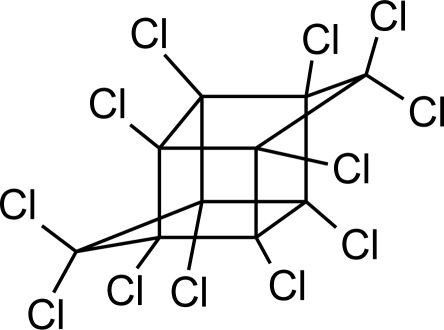	Weak estrogen effect [13]	M.P: 0.2–1.5 ng/g lipid [94] B.S: <LOD–7.2 ng/g lipid [95] H.M: 0.2–1.7 ng/g lipid [98]
**Molinate (H)** M(g/Mol) = 187.3 pKa = n.a logP: 2.86	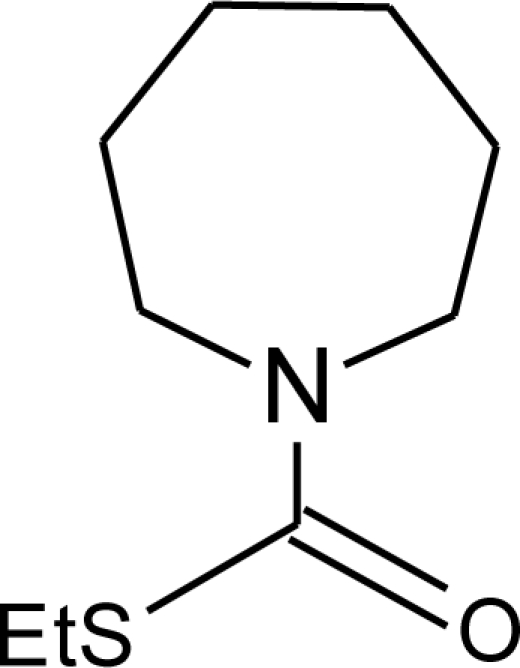	Reproductive tract damage, reduction of fertility [13]	
**Myclobutanil (F)** M(g/Mol) = 288.8 pKa = 2.3 logP: 2.89	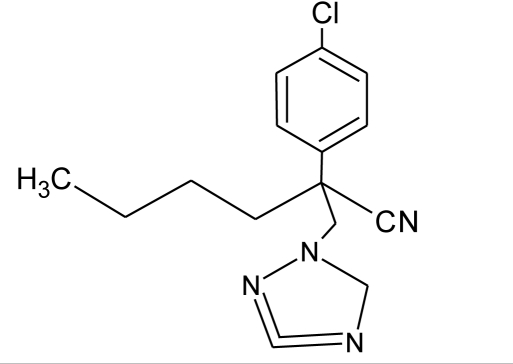	Weak estrogen and androgen inhibition, Binding to estrogen and androgen receptors, aromatase inhibition [89,90,119]	
**Nitrofen (H)** M(g/Mol) = 284.1 pKa = n.a logP: 3.4	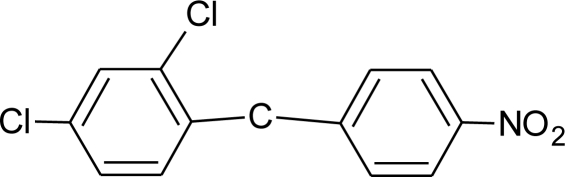	Estrogen and androgen inhibition [90]	
**Oxamyl (I)** M(g/Mol) = 219.3 pKa = n.a logP: −0.44	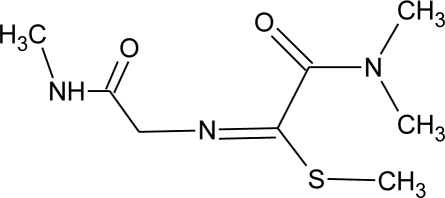	Weak estrogen effect [13]	
**Parathion (I)** M(g/Mol) = 291.3 pKa = n.a logP: 3.83	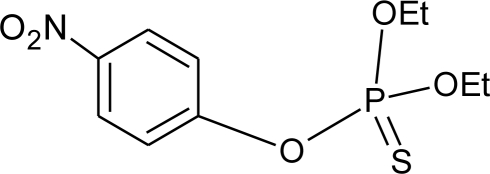	Inhibition of catecholamine secretion, increase of melatonin synthesis, inhibition of gonadotrophic hormone [13]	U: <LOD–84 ng/mL * [68]
**Penconazole (F)** M(g/Mol) = 284.2 pKa = 1.51 logP: 3.72	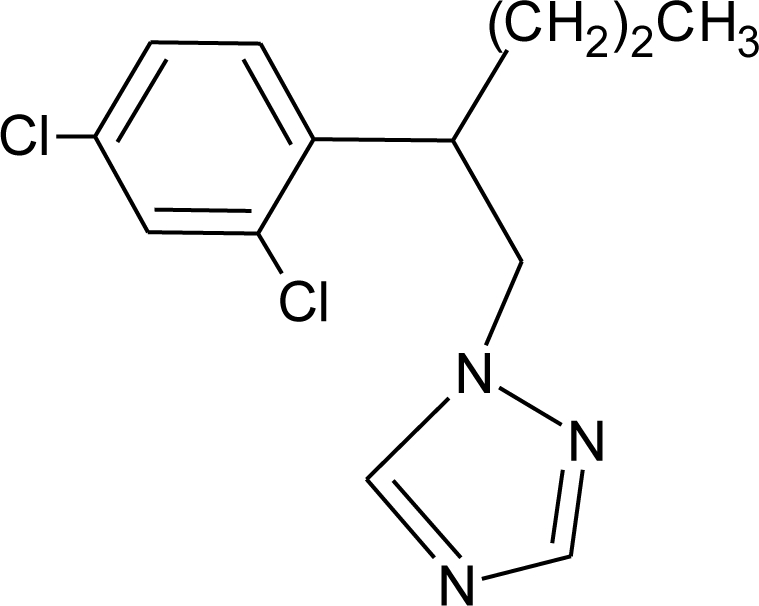	Weak estrogenic effect. Inhibition of aromatase activity, decrease of estrogens production and increase androgens availability [89,119]	
**Pentachlorophenol (H, F, I)** M(g/Mol) = 266.3 pKa = 4.73 logP: 3.32	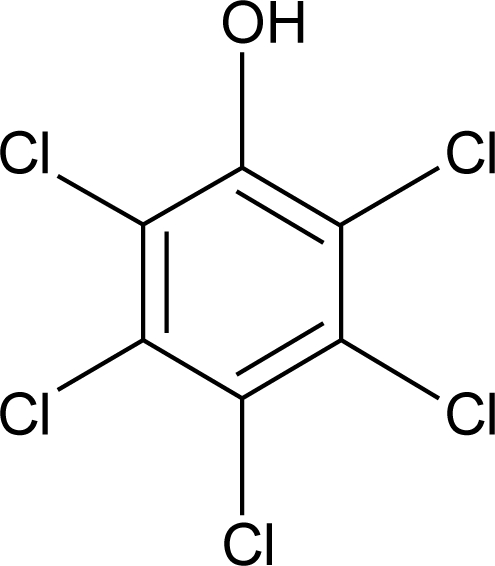	Weak estrogenic and anti-androgenic affect [13]	A.F : 0.15–0.54 ng/mL [135]
**Permethrin (I)** M(g/Mol) = 391.3 pKa = n.a logP: 6.1	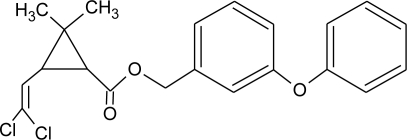	Inhibition of estrogen-sensitive cells proliferation [87,106]	U: 1–150 μg/g * [107]
Phenylphenol (F) M(g/Mol) = 170.2 pKa = 9.97 logP:3.09	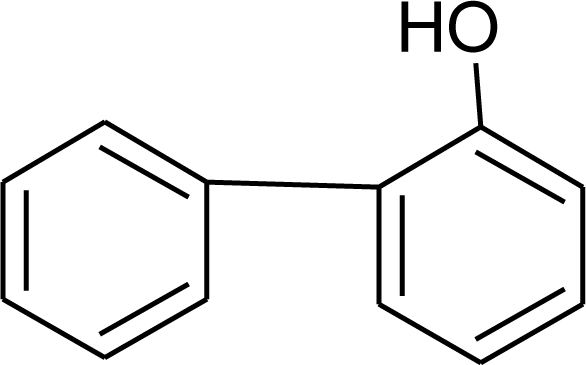	Estrogen agonist [136]	A.F: 0.1–0.17 ng/mL [135]
**Prochloraz (F)** M(g/Mol) = 376.7 pKa = 3.8 logP: 3.53	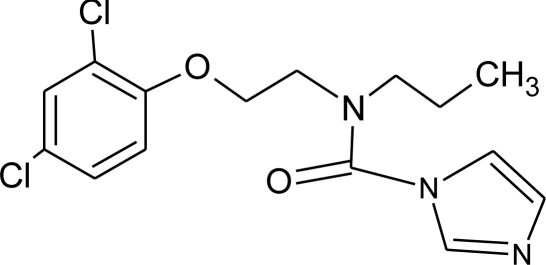	Activation of Pregnane X cellular receptor. Antagonist to cellular androgen and estrogen receptors, agonist to Ah receptor and inhibition of aromatase activity [8,11,120,137]	
**Procymidone (F)** M(g/Mol) = 284.1 pKa = n.a logP: 3.3	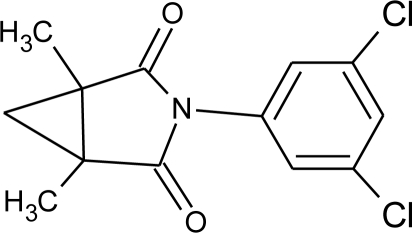	Competitive binding to androgen receptor [131]	
**Propamocarb (F)** M(g/Mol) = 188.3 pKa = 9.5 logP: 0.84	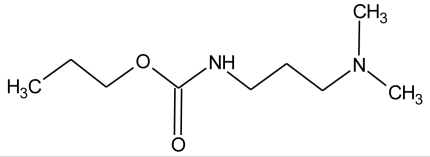	Weak increase of aromatase activity and estrogen production [8]	
**Propanil (H)** M(g/Mol) = 318.1 pKa = n.a logP: 2.29	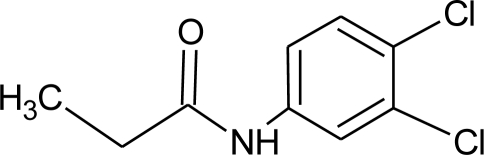	Increase of cellular response to estrogen [138]	
**Propazine (H)** M(g/Mol) = 229.8 pKa = 1.7 logP: 3.95	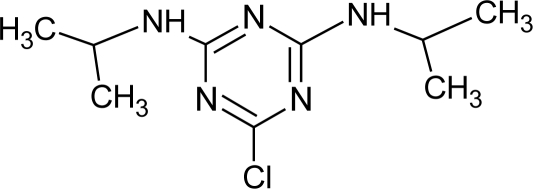	Induction of aromatase activity and increase of estrogen production [81]	
**Propiconazole (F)** M(g/Mol) = 342.2 pKa = 1.09 logP: 3.72	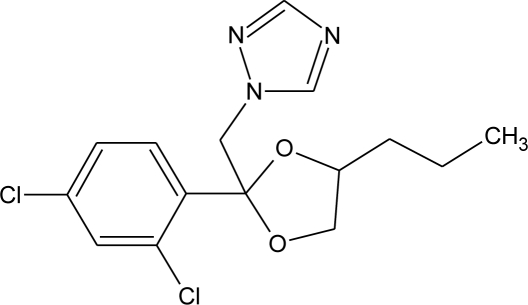	Weak estrogen and aromatase activity inhibition. Decrease estrogens production and increase of androgens availability [89,119]	
**Propoxur (I)** M(g/Mol) = 209.2 pKa = n.a logP: 0.14	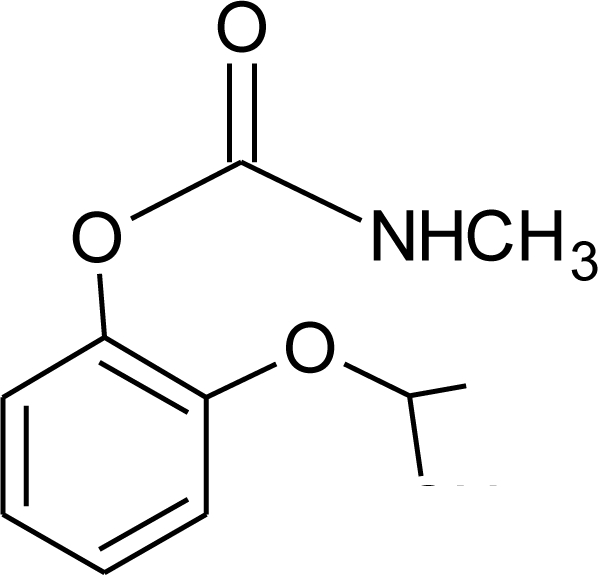	Weak estrogenic effect [13]	M: 0.24–1.50 μg/mL [88] C.B: 0.77 μg/mL [88] H: 0.22–0.42 μg/mL [88] M.B: 0.67–0.77 μg/mL [88]
**Prothiophos (I)** M(g/Mol) = 345.3 pKa = n.a logP: 5.67	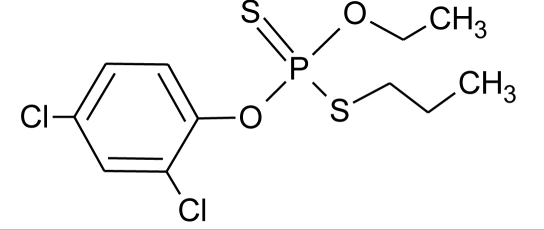	Estrogenic effect [115]	
**Pyridate (H)** M(g/Mol) = 378.9 pKa = n.a logP: 0.5	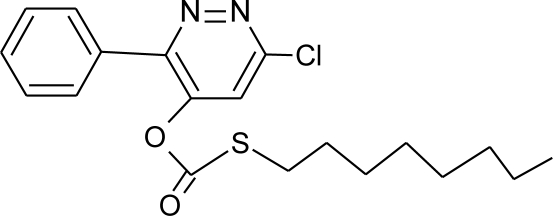	Binding to estrogen and androgen receptors [90]	
**Pyrifenox (F)** M(g/Mol) = 295.2 pKa = 4.61 logP: 3.4	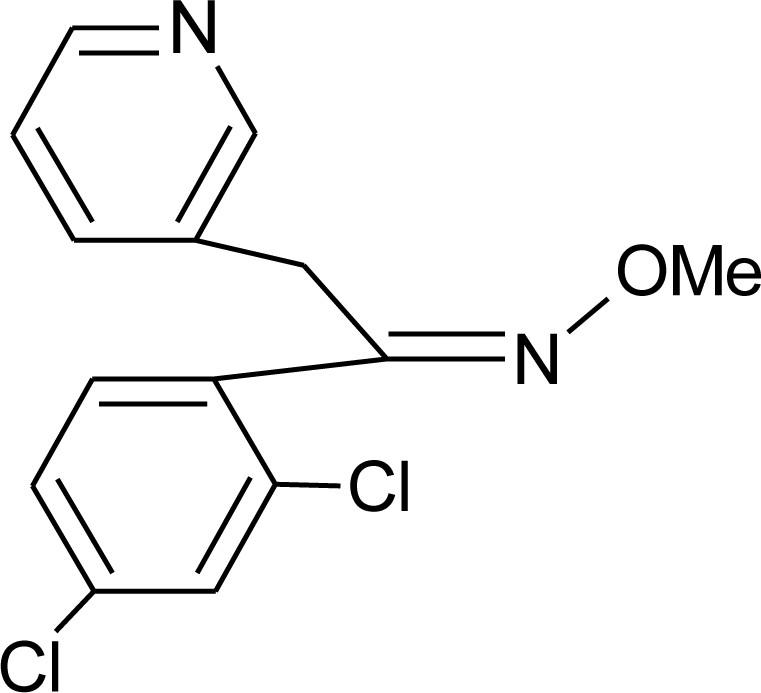	Weak estrogen inhibition [119]	
**Pyripyroxifen (I)** M(g/Mol) = 321.4 pKa = 6.87 logP: 5.37	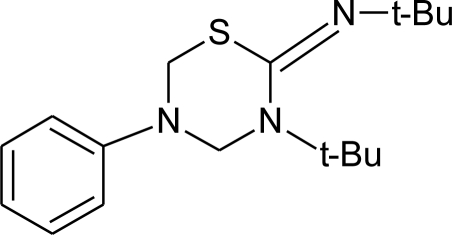	Estrogenic effect [115]	
**Resmethrin (I)** M(g/Mol) = 338.4 pKa = n.a logP: 5.43	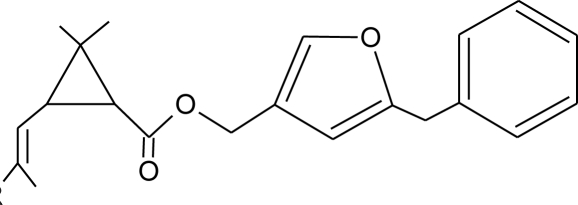	Binding to sex hormone [124]	
**Simazine (H)** M(g/Mol) = 201.7 pKa = 1.62 logP: 2.3	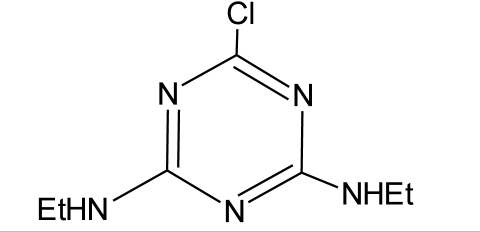	Induction of aromatase activity, increase of estrogen production [81]	
**Sumithrin (I)** M(g/Mol) = 350.5 pKa = n.a logP: 6.01	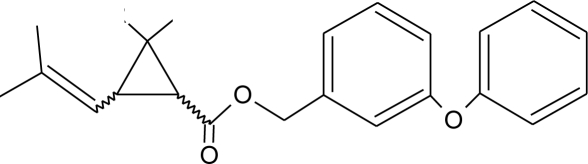	Increase of estrogen-sensitive cells proliferation, antagonist of the progesterone action [87,123]	
**Tebuconazole (F)** M(g/Mol) = 307.8 pKa = n.a logP: 3.7	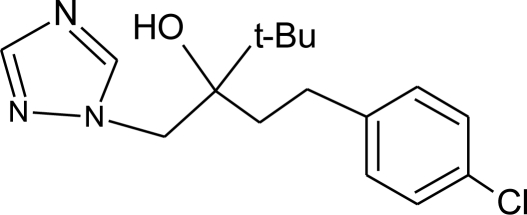	Inhibition of aromatase activity, decrease the estrogens production and increase androgens availability [89]	
**Tetramethrin (I)** M(g/Mol) = 331.4 pKa = n.a logP: 4.6	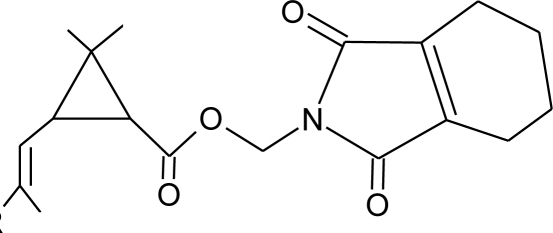	Estrogen-antagonistic effects in females only [139]	
**Tolchlofos-methyl (I)** M(g/Mol) = 301.1 pKa = n.a logP: 4.56	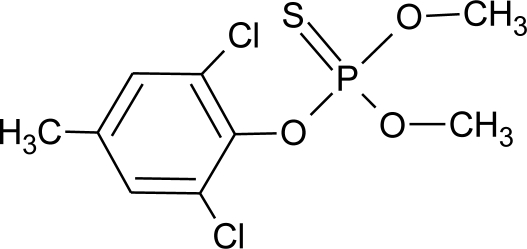	Competitive binding to cellular estrogen receptors [120]	
**Toxaphene (I)** M(g/Mol) = 411.8 pKa = n.a logP: 3.3	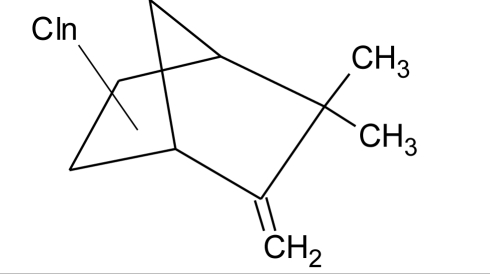	Increase of estrogen-sensitive cells proliferation. Inhibition of corticosterone synthesis in the adrenal cortex [13,116]	
**Triadimefon (F)** M(g/Mol) = 293.8 pKa = n.a logP: 3.18	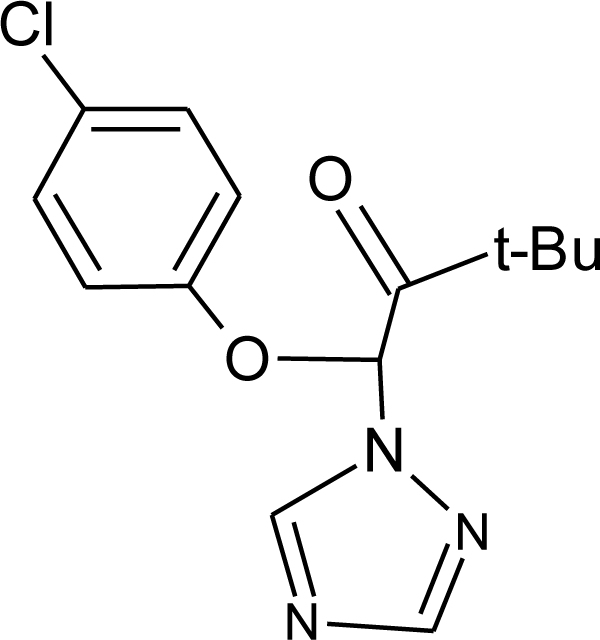	Estrogenic effect, inhibition of aromatase activity, decrease of estrogens production and increase androgens availability [90]	
**Triadimenol (F)** M(g/Mol) = 295.8 pKa = n.a logP: 3.18	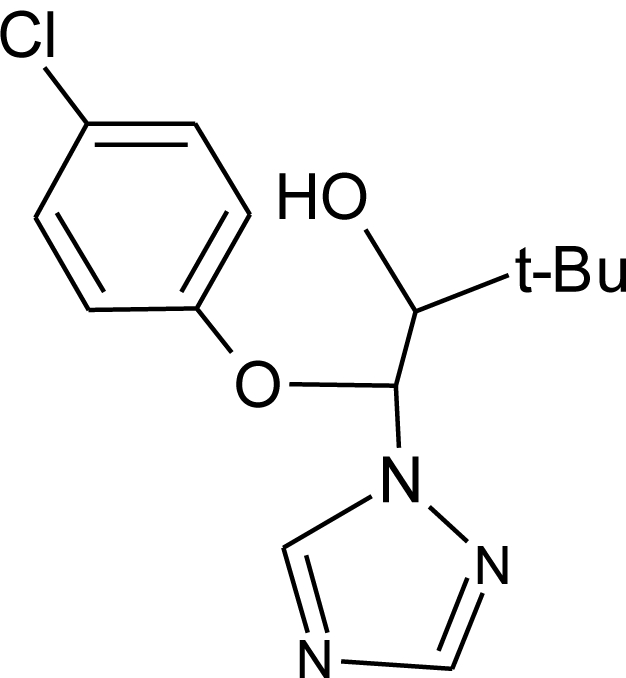	Estrogenic effect, inhibition of aromatase activity, decrease of estrogens production and increase androgens availability [89,90]	
**Tribenuron-methyl (H)** M(g/Mol) = 395.4 pKa = 4.7 logP: 0.78	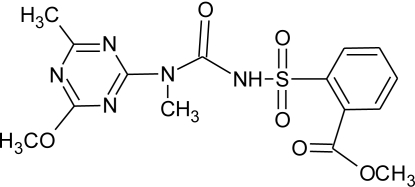	Weak estrogenic effect [8]	
**Trichlorfon (I)** M(g/Mol) = 257.4 pKa = n.a logP: 0.43	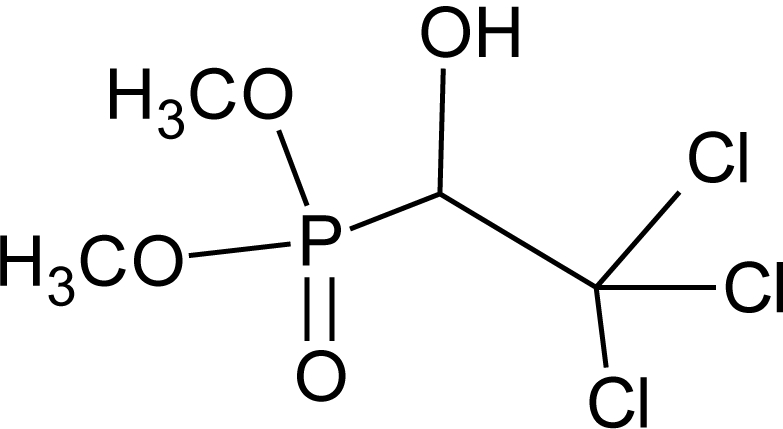	Alteration of thyroid function [140]	
**Trifluralin (H)** M(g/Mol) = 335.3 pKa = n.a logP: 5.27	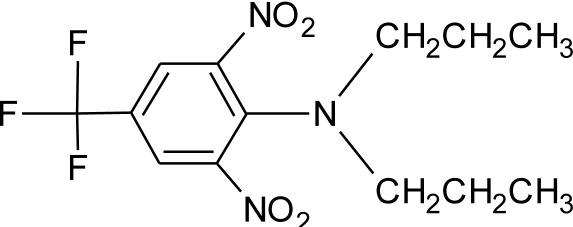	Interaction withs pregnane X cellular receptor, interference steroid hormone metabolism [74]	M.S: 0.00–8.5 ng/g [92] U.C: 0.007–4.42 ng/g [92]
**Vinclozolin (F)** M(g/Mol) = 286.1 pKa = n.a logP: 3.02	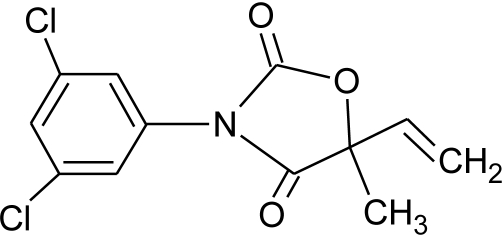	Competitive binding to androgen receptor Interactions with pregnane X cellular receptor, interference with steroid hormone metabolism. [8,74,131]	

(H): Herbicide, (F): Fungicide, (I): Insecticide, (AFA): Antifouling agent, (T): Termiticide, U: urine, S: semen, H.S: human serum, H.M human milk, M: mecomium, H = hair, A.T: adipose tissues, F.F: follicular fluid, M.P: maternal plasma, U.C: umbilical cord.

* Measured by the presence of its metabolite.

** Case of poisoning patient.

## Abbreviations:

ADI: Acceptable Daily Intake;
AhR: ArylHydrocarbon Receptor;
AR: Androgen Receptor;
CA: Concentration Addition;
CAR: Constitutive Androstane Receptor;
EDC: Endocrine Disruptor Chemical;
ER: Estrogen Receptor;
ERR: Estrogen Related Receptor;
HCH: Hexachlorocyclohexane;
IA: Independent Action;
LOD: Limit of Detection;
PCB: PolyChloroBiphenyl;
PXR: Pregnane X Receptor;
WHO: World Health Organisation
